# Mother’s knowledge on prevention of mother-to-child transmission of HIV, Ethiopia: A cross sectional study

**DOI:** 10.1371/journal.pone.0203043

**Published:** 2018-09-11

**Authors:** Yihun Mulugeta Alemu, Tesfa Dejenie Habtewold, Sisay Mulugeta Alemu

**Affiliations:** 1 School of Public Health, College of Medicine and Health Science, Bahir Dar University, Bahir Dar, Ethiopia; 2 Department of Epidemiology, University Medical Center Groningen, University of Groningen, Groningen, The Netherlands; 3 International Medical Corps, Mental Health and Psychosocial Support Program, Dollo Ado Refugee Camp, Dollo Ado, Ethiopia; Ohio University Heritage College of Osteopathic Medicine, UNITED STATES

## Abstract

**Objective:**

To identify proportion of and factors for comprehensive knowledge on prevention of mother-to-child transmission of HIV in pregnant women attending antenatal care in Northern Ethiopia.

**Methods:**

A total of 416 pregnant women were interviewed between October 2012 and May 2013. Logistic regression analysis was used to identify factors for comprehensive knowledge on prevention of mother-to-child transmission of HIV.

**Results:**

The proportion of pregnant women, who have comprehensive knowledge on prevention of mother-to-child transmission of HIV, was 52%. The odds of having comprehensive knowledge on prevention of mother-to-child transmission of HIV were higher among pregnant women who were younger (16 to 24 years old) (Adjusted Odds Ratio (AOR) = 2.95; 95%CI: 1.20, 7.26), urban residents (AOR = 2.45; 95%CI: 1.39, 4.32), attending secondary education and above (AOR = 4.43; 95%CI: 2.40, 8.20), employed (AOR = 4.99;95%CI: 2.45, 10.16), have five children or more (AOR = 9.34; 95%CI:3.78, 23.07), have favored attitude towards HIV positive living (AOR = 2.53; 95%CI: 1.43, 4.44) and have perceived susceptibility to HIV (AOR = 10.72; 95%CI: 3.90, 29.39).

**Conclusion:**

The proportion of women who have comprehensive knowledge on prevention of mother-to-child transmission of HIV in this study setting was low. Measures which will escalate mother’s knowledge on prevention of mother-to-child transmission of HIV should be emphasized. Efforts to improve mother’s knowledge on prevention of mother-to-child transmission of HIV should target women who were older age (> = 35years), rural residents, unemployed, not attending formal education, primigravids, have no favored attitude towards HIV positive living and have not perceived susceptibility to HIV.

## Background

Globally, significant numbers of people are living with HIV. More than 90 percent of new pediatric HIV infections are in sub-Saharan Africa [1]. In Ethiopia, an enormous number of children acquires HIV infection every year [2]. Mother–to–child transmission (MTCT) is the most common means of acquiring pediatric HIV infection since more than 90% of new HIV infection among children is through mother-to-child transmission. Without any intervention measures to prevent the transmission, the risk of MTCT ranges from 20% to 40%. However, mother-to-child transmission can be reduced to less than 2% in non–breastfeeding populations. In breast feeding populations, the transmission can be reduced less than 5% with effective interventions during the periods of pregnancy, labor, delivery and breastfeeding [3].

Prevention of mother-to-child transmission (PMTCT) is one of the fundamental approaches to control HIV epidemic [1]. In early 2013, Ethiopia launched option B+ implementation; i.e. all HIV infected pregnant women receive triple ARV (anti-retro viral) drugs without an initial CD4 testing [4]. To control the risk of MTCT of HIV, World Health Organization has launched a program as virtual elimination of pediatric HIV. Four-pronged approaches are incorporated as components of virtual elimination of pediatrics’ HIV. The approaches include primary prevention of HIV infection among women of childbearing age; preventing unintended pregnancies among women living with HIV; preventing HIV transmission from a woman living with HIV to her infant and providing appropriate treatment, care and support to mothers living with HIV, their children and families [3]. For implementing prevention of mother-to-child transmission of HIV, one of the major problems is poor awareness and knowledge of the people about MTCT and PMTCT. Particularly, mother’s knowledge on PMTCT plays significant roles to realize preventive measures and to utilize the service [5].

Mother’s knowledge on prevention of mother-to-child transmission of HIV is essential in order to use available prevention options [6]. Women, who have adequate knowledge on HIV prevention, protect themselves, their husband and their children from HIV infection and are more likely to undergo HIV testing than women who do not have adequate knowledge on HIV [7]. On the other hand, women, who do not realize mother-to-child transmission of HIV and its prevention, have limited uptake of PMTCT services [8]. Regardless of widespread extension of PMTCT services, women’s knowledge on PMTCT is not satisfactory [9,10]. Investigating the proportion and predictors for mother’s knowledge on prevention of mother-to-child transmission of HIV in resource limited settings have many benefits. It is a critical requirement to enhance mother’s knowledge on PMTCT. The investigation will also help to escalate utilizations of PMTCT services and it will ultimately be used to prevent and control transmission of HIV.

Previous studies have identified factors for mother’s knowledge on prevention of mother-to-child transmission of HIV [11–16]. However, different levels of mother’s knowledge on PMTCT were observed [17–20]. Contributions of factors for mother’s knowledge on PMTCT vary in settings of different populations [19–25]. We aim to identify proportion of and factors for comprehensive knowledge on prevention of mother-to-child transmission of HIV among pregnant women in Northern Ethiopia.

## Methods

### Study design and study settings

We conducted a facility based cross sectional study from October 2012 to May 2013 among pregnant women attending antenatal care in East Gojjam, Northern Ethiopia. In Northeast Gojjam Zone, 18 governmental health institutions are providing primary healthcare. Antenatal care service is provided in all primary healthcare centers. All mothers, who come for antenatal care, are linked to integrated PMTCT services [6]. Nurses, Midwifes and Health Officers are providing counselling and other four-pronged PMTCT services. Four governmental health institutions in the four districts were included (Motta, Gedieweyne, Debrewerk and Bichena).

### Sample size estimation

Sample size was estimated using a single population proportion estimation formula and calculated by using Epi-info 7 with 50% proportion, 5% of absolute precision, 95% confidence interval and non-response rate of 10%. The calculated overall sample size was 422. The sample size was calculated for different objectives. We took the largest estimated sample.

### Sampling procedures

From 18 primary healthcare centers in Northeast Gojjam Zone, four primary healthcare centers (Motta, Gendowin, Bitchena and Debrewerk) were selected by using lottery method and therefore included in the study. Pregnant women, who were attending the first antenatal care visit of their current pregnancies, were recruited. Systematic random sampling with sampling interval of three was used to select pregnant women from each health institution.

### Data collection and analysis

Questionnaires were first prepared in English. It was then translated to Amharic and back translated to English. Pretest was conducted for consistency and ease of understanding. The study questionnaire was pretested in similar settings to the study area for two times. Firstly, pretest was conducted at Bahir Dar Zuria Werda primary healthcare center, West Gojjam Zone, among 20 pregnant women attending the first antenatal care of their current pregnancy; in this case, the questionnaire was validated for easy of communication. Vague words, phrases and sentences were corrected. Secondly, after the first pretest, study questionnaire was re-tested in Meray Woreda primary healthcare center, West Gojjam Zone, in 20 pregnant women attending the first antenatal care of their first pregnancy to endorse consistency of understanding among respondents; almost all respondents fully understand the questions. The final version of survey questionnaire was used to collect the actual data.

Data were collected from pregnant women during their first antenatal care visit. Trained nurses conducted face to face interviews with study participants by using study questionnaires. Data were collected to assess comprehensive knowledge on prevention of mother-to-child transmission of HIV and its associated factors (age, education, residence, employment, perceived susceptibility to HIV and attitude to HIV positive living).

Data were entered into Epi info 7 and analysis was done by using STATA 12. Frequencies and proportions were used to describe the study subjects in relation to the studied variables. Logistic regression model was used to examine the relation between explanatory variables and comprehensive knowledge on prevention of mother-to-child transmission of HIV. Bi-variable logistic regression models were fitted for all explanatory variables. Odds ratio with 95% confidence interval and p-value were used to measure strength of association and to identify statistical significance of results. Identification of confounding variables by using logistic regression model was used; predictor such as health institution and other covariates were adjusted for confounding. P-value less than 0.2 was used as a cutoff point to include explanatory variables from bi-variable models into a multivariable model. In this data set all explanatory variables that were fitted in the bi-variable model were fitted to a multivariable logistic regression model.

The Hosmer Lemeshow test was applied and the fit of the model was checked: a poor fit of the model if the p value is < 0.05 and good fit if the p-value is > 0.05. In this study the model was adequately fitted the data and the p-value was 0.93. The clustered nature of the data was taken into account in the analysis; however, multilevel logistic regression model was not fitted. Hence, the ICC (intra-class correlation coefficient) estimate of these data was 0.006 indicating that only 0.6% of the variation is due to difference between health institutions. Most of the variations (99.4%) were explained by the lower level measures (pregnant women). As Intra-class correlation coefficient was less than 5–10%, hierarchical modeling is not required [26].

### Operational definitions

#### Comprehensive knowledge of PMTCT

Pregnant women were classified as knowledgeable if the women knew at least one means mother-to-child transmission of HIV (during pregnancy, delivery or breast feeding) and method of prevention from mother-to-child transmission of HIV (antiretroviral therapy for the mother and for the baby).

#### Favorable attitude towards persons living with HIV

Defined as a woman who perceived that her husband or/ and other family member/s will care for her if her HIV test result is positive. The following question was asked. If your result turns out to be positive, what would be the likely reaction to your husband or relatives? The possible answers were four; no one will believe the results; I will be thrown out of home; I will be physically violated/abused; or he/they will start to care for me.

#### Perceived susceptibility to HIV

Pregnant women who perceive the risk of acquiring HIV infection. The following question was asked. Do you think, you have risk of acquiring HIV? The possible answer was Yes or No.

### Ethical considerations

Ethics approval was received from Bahir Dar University College of Medicine and Health Science research and ethical review committee. Written permission to conduct the study was gotten from each health institution involved in the study. In addition, informed written consents were obtained from study participants whose age were 18 years and more. This study also included participants between 16 years of age and 18 years; therefore, written assents from the teenagers and written permissions from their parents were done. Since there were illiterate participants and parents, the data collectors informed participants and parents about informed consents and assents. Willingness to participate in the study and parental permission were confirmed by signing (finger print for those who can’t sign) on the informed consent sheet.

## Results

### Demographic and perceptions of study participants

A total of 416 pregnant mothers were included in the analysis. The mean age and Standard Deviation (SD) of study participants was 28.2 years (SD: 6.15 years).

Table 1 presents frequency distribution of factors for comprehensive knowledge on prevention of mother-to-child transmission of HIV. The majority of pregnant women (55%) came from rural area. More than half of study participants (54%) were not attended formal education. Higher proportion of mothers (63%) didn’t have favored attitude towards HIV positive living. Only 15% pregnant women have perceived their risk of acquiring HIV infection.

**Table 1:** Study variables for comprehensive knowledge of mother-to-child transmission of HIV in pregnant women attending antenatal care in East Gojjam, Northern Ethiopia, 2013.

Variables	No CKPMTCT n (%)	Have CKPMTCT n (%)	Total (%)
**Health Institution’s site**			
Motta	86 (48.3%)	92(51.7%)	178(42%)
Gndweyene	35 (47.9%)	38(52.1%)	73(17.6%)
Debrewerke	37(48.1)	40(51.9)	77(18.5%)
Bichena	42 (47.7)	46(52.3)	88(21.3%)
**Age**			
16–24	50(41)	72(59)	122(29)
25–29	57(43)	76(57)	133(32)
30–34	40(46)	47(54)	87(21)
> = 35	53(71)	21(29)	74(18)
**Residence**			
Rural	146(64)	83(36)	229(55)
Urban	54(29)	133(71)	187(45)
**Education**			
No formal education	153(69)	70(32)	223(54)
Primary education	10(26)	28(74)	38(10)
Secondary and above	37(24)	118(50)	155(37)
**Occupation**			
Unemployed	184(62)	115(38)	299(72)
Employed	16(14)	101 (86)	117(38)
**Parity**			
0	89(74.2)	31(25.58)	120(29)
1–4	98(45.2)	119(54.8)	217(52)
≥ 5	13(16.5)	66(83.5)	79(19)
**Favored attitude towards HIV positive living**			
**Yes**	44(28)	115(72)	155 (37)
No	156(61)	101(39)	266 (63)
**Perceived susceptibility to HIV**			
Yes	9(15)	53(85)	62(15)
No	191(64)	163(36)	354(85)

CKPMTCT = comprehensive knowledge on mother-to-child transmission of HIV

### Proportion of comprehensive knowledge on prevention of mother-to-child transmission of HIV

In this study 52% of pregnant women had comprehensive knowledge on prevention of mother-to-child transmission of HIV. Higher proportion of younger age (16 to 24 years) women (59%) had comprehensive knowledge on prevention of mother-to-child transmission of HIV. Similarly, majority of women (85%), who perceived their risk of acquiring HIV infection, had comprehensive knowledge on prevention of mother-to-child transmission of HIV.

Table 2 shows factors for comprehensive knowledge on prevention of mother-to-child transmission of HIV. Younger age (16 to 24 years old) (AOR = 2.95; 95%CI: 1.20, 7.26) was an independent factor for increased comprehensive knowledge on prevention of mother-to-child transmission of HIV compared with women older than 35 years. Urban residency (AOR = 2.45; 95%CI: 1.39, 4.32) was an independent factor for increased comprehensive knowledge on prevention of mother-to-child transmission of HIV. Similarly, women who attended secondary education and above (AOR = 4.39; 95%CI: 2.), were more likely to have higher comprehensive knowledge on prevention of mother-to-child transmission of HIV than women who do not attend formal education. Employed women (AOR = 4.99;95%CI: 2.45, 10.16) were more likely to have increased comprehensive knowledge on prevention on mother-to-child transmission of HIV than unemployed women. Likewise, having five or more children (AOR = 9.34; 95%CI:3.78, 23.07), favored attitude towards HIV positive living (AOR = 9.34; 95%CI:3.78, 23.07) and perceived susceptibility to HIV (AOR = 11.72; 95%CI: 3.90, 29.39) were independently associated with increased comprehensive knowledge on prevention of mother to child transmission of HIV among pregnant women.

**Table 2:** Factors independently associated with comprehensive knowledge on prevention of mother-to-child transmission of HIV in pregnant women, Northern Ethiopia, 2013.

Variable	COR(95%CI)	P-value	AOR(95%CI)	P-value
**Age**				
16–24	3.63(1.95,6.76)	< 0.001	2.95(1.20, 7.26)	0.018
25–29	3.36(1.92,6.20)	< 0.001	2.49(1.06, 5.86)	0.036
30–34	2.96(1.53, 5.72)	0.001	3.18(1.24, 8.13)	0.016
> = 35	1		1	
**Residence**				
Rural	1		1	
Urban	4.33(2.86, 6.56)		2.45(1.39,4.32)	0.002
**Education**				
No formal education	1		1	
Primary education	6.12(2.81, 13.29)	< 0.001	3.35(1.32, 8.48)	0.0113
Secondary above	6.97(4.37, 11.09)	< 0.001	4.43(2.40, 8.20)	< 0.001
**Occupation**				
Unemployed	1		1	
Employed	10.10(5.67, 17.97)	< 0.001	4.99(2.45,10.16)	< 0.001
**Parity**				
0	1			
1–4	3.48(2.13,5.68)	< 0.001	2.69(1.36, 5.30)	0.004
≥ 5	14.57(7.08, 29.99)	< 0.001	9.54(3.90, 23.32)	< 0.001
**HIV positive living**				
Favored attitude	4.03(2.63, 6.19)	< 0.001	2.53(1.43,4.44)	0.001
Not favored attitude	1		1	
**Perceived susceptibility to HIV**				
Yes	6.9 (3.30, 14.41)	< 0.001	11.72(3.9, 29.39)	< 0.001
No	1		1	

## Discussion

This study described the proportion of mother’s knowledge on PMTCT. In addition, this study identified predictors for mother’s knowledge on PMTCT. The proportion of mothers, who do have comprehensive knowledge, was low. These findings have important impact on public health so as to prevent and control transmission of HIV particularly in lower income settings, where HIV overwhelm the already limited health system. The study findings should alert public health agencies since mother’s comprehensive knowledge on PMTCT is still unsatisfactory. Furthermore, as this study was a preliminary assessment, researchers should explore other means that can potentially enhance mother’s knowledge on PMTCT.

In this research 52% of pregnant women had comprehensive knowledge on prevention of mother-to-child transmission of HIV. This finding is consistent with a study conducted in Tanzania which described the proportion of women, who have adequate knowledge of PMTCT, was 46% [21]. However, our finding was higher than a study conducted in Gambila region which showed that only 17% pregnant women knew prevention of mother-to-child transmission of HIV [27]. Variability of mother’s knowledge on prevention of mother-to-child transmission of HIV was observed among studies. The proportions of mother, who do have adequate knowledge on preventions of mother-to-child transmission of HIV, rang from 9% to 78% [17–19, 27,28].The variations in proportions of mother’s knowledge among studies could be due to differences in study periods. Increased proportions of women, who have comprehensive knowledge on prevention of mother-to-child transmission of HIV, have observed in recent times. Furthermore, difference in source population may be linked with difference on factors that affect mother’s knowledge on preventions of mother-to-child transmission of HIV.

In our study, younger age was an independent factor for increased comprehensive knowledge on prevention of mother-to-child transmission of HIV. It was consistent with a research finding that showed older age women had lower level of knowledge on PMTCT [20]. This could be due to opportunities among younger women, who have better access to education, enable them to have more information sources such as newspaper and social media. In contrast, a study conducted in Kenya showed that there was no statistically significant difference between knowledge of teenage pregnant women compared with older pregnant women [14]. This could be due to smaller sample size of the study conducted in Kenya that lower the power of the study to detect the association.

Similar to studies conducted in Tanzania and Sudan [15,16]. Our study identified that women, who live in urban area, were more likely to have knowledge on prevention of mother-to-child transmission of HIV compared with women who live in rural area. Most mothers, who live in rural area, had limited access to PMTCT service. Even if some women do have accesses to PMTCT services, their services utilization remains low. Being a rural resident was found to be a barrier for uptake of PMTCT services [22–24]. Therefore, knowledge of the mother on PMTCT could be affected by access and utilizations of the services. Mothers, who are attending PMTCT services, also obtain PMTCT knowledge and counseling [4]. Correspondingly, knowledge of the mother attributes to utilization of PMTCT services. Pregnant women, who do have comprehensive knowledge on prevention of mother-to-child transmission, are more likely tested for HIV than women who do not have comprehensive knowledge on prevention of mother-to-child transmission of HIV [7].

Mothers in this study, who attended formal education, were more likely to have higher knowledge on prevention of mother-to-child transmission of HIV than mothers who do not attend formal education. Level of education is linked with understanding of HIV/AIDS transmission and its prevention. Populations, who attended formal education, have demonstrated adequate understanding of HIV transmission and its prevention [29]. Limited knowledge on PMTCT, on the other hand, was identified in pregnant women who had lower level of education [30]. Similarly, low level of educational was associated with deceased uptake of PMTCT services [25]. Missed opportunities of PMTCT in health facilities as well as in community engagement services were higher in pregnant women with low level of education [31]. Maternal knowledge about PMTCT could directly be affected by school training that enables mothers to understand diseases transmission and its prevention. Educated mothers are privileged in accessing and utilizing PMTCT services that enhance mother’s knowledge on PMTCT [7].

This research finding revealed that unemployed women were less likely to have knowledge on prevention of mother-to-child transmission of HIV than employed women. Often, employed women are advantageous in terms of income and social networking than unemployed. Income-level of the mother is linked with the mother’s knowledge on preventions of mother-to-child transmission of HIV [32]. Acquiring of HIV related knowledge is associated with income-level in the way that higher income group have better access of information and services on PMTC [33, 34]. Employed women are connected with higher income level; employment was coupled with access and use of favored social-networking. Employment enables women to have social links with groups who do have better information on HIV/AIDS. Knowledge is shared within group members or among different groups. Among working groups, interchange of ideas and learning about health services were evidenced in various types of working environments [35–37].

Women who have good knowledge on HIV/AIDS such as adequate knowledge on antiretroviral therapy and on HIV positive living, are able to cope stigma, discrimination and stereotype [37]. Likewise, favored attitude towards HIV positive living was independently associated with increased knowledge on prevention of mother-to-child transmission of HIV. Perceived susceptibility to HIV was associated with increase awareness of HIV services and higher utilizations of health services. On the other hand, those who don’t have adequate knowledge on HIV believed that they don’t have the risk of acquiring HIV infection once they are married. Nevertheless, newly HIV infected pregnant women were identified among married couples in similar settings [38].

### Limitations

Since this research was health institution based cross-sectional study, the data can’t be inferred to the general population. The findings can’t extrapolate to pregnant women who didn’t attend the health institutions. Since temporal relationship of outcome and exposure variables is hardly possible, this study does not establish causations. However, the multivariable analysis indicated strong associations between exposure and outcome variables; therefore, this study provides valuable information that will enhance mother’s knowledge on prevention of mother-to-child transmission of HIV.

## Conclusion

Proportion of women, who have comprehensive knowledge on prevention of mother-to-child transmission of HIV in this study, was low. Measures, which will escalate mother’s knowledge on prevention of mother-to-child transmission of HIV, should be emphasized. Efforts to improve mother’s knowledge on prevention of mother-to-child transmission of HIV should target women who are older age (> = 35years), live in rural, unemployed, not attending formal education, primigravids, have no favored attitude towards HIV positive living and have not perceived susceptibility to HIV.

## Supporting information

S1 File This is the English version of study questionnaire. This is a copy of English Version Study questionnaire.
(DOCX)

S2 File This is the Amharic version of study questionnaire. This is a copy of Amharic Version Study questionnaire.
(DOCX)

S3 File This is all relevant data of this study. This is the de-identified data.
(XLSX)
