# Supplementary material for: Mother’s knowledge on prevention of mother-to-child transmission of HIV, Ethiopia: A cross sectional study
Source: PLoS One. 2018 Sep 11;13(9):e0203043. doi: 10.1371/journal.pone.0203043 (PMC6133350; doi:10.1371/journal.pone.0203043)
Supplement: S2 File — This is a copy of Amharic Version Study questionnaire. (DOCX) [file pone.0203043.s002.docx]

**ተጨማሪ፡ መጠይቅ**

አጠቃላይ መረጃ

|  | የጥያቂ ዐይነት | መልስ | ኮድ | ዕለፍ |
| --- | --- | --- | --- | --- |
| 01 | ጢና ተቆም | ሞጣ  ግንድ ወይን  ቢቸና  ደብረ ወርቅ | 1  2  3  4 |  |

ክፍል ዐንድ: ሕብረተሰባዊ አና ውልደታዊ መረጃ

|  | የጥያቂ ዐይነት | መልስ | ኮድ | ዕለፍ |
| --- | --- | --- | --- | --- |
| 02 | መኖርያ | ከተማ  ገጠር | 1  2 |  |
| 03 | እድሚ |  |  |  |
| 05 | የልጆች ብዛት |  |  |  |
| 07 | የትምርት ደርጃ | መደበኛ ትምህርት ያላተማረ  የመጀመሪያ ደርጃ (1-8)  ሑለተኛ ደርጃ /9-12  ኮሊጅ /ዩንቨርስቲ | 1  2  3  4 |  |

| 8 | ሰራ | ያለው  የሊለው | 1  2 |  |
| --- | --- | --- | --- | --- |
| 9 | የወር ገቢ | ከ 30 ዶላር ያነሰ  ከ 30 ዶላር በላይ | 1  2 |  |

ክፍል ሑለት: የኢች አይቪ እውቀት እና አስተሳሰብ

| ቁ | የጥያቂ ዐይነት | መልስ | ኮድ | ዕለፍ |
| --- | --- | --- | --- | --- |
| 1 | ኢች አይቪ መተላለፍያ መንገዶች | ግብረስጋ ግንኙነት | 1 |  |
|  |  | በደም አና በደም ንክኪ | 2 |  |
|  |  | ከእናት ወደ ልጅ | 3 |  |
|  |  | በሰለታማ ነገሮች | 4 |  |
|  |  | አላውቀውም | 5 |  |
|  |  | ሊላ (ይጠቀስ) |  |  |
| 2 | ኢች አይቪ መከላካያ መንገዶች | መቆጠብ | 1 |  |
|  |  | መታምን | 2 |  |
|  |  | ኮንዶም | 3 |  |
|  |  | አላውቀውም | 4 |  |
|  |  | ሊላ (ይጠቀስ) |  |  |
| 3 | ኢች አይቪ ከእናት ወደ ልጅ ይተላለፋል | አወ | 1 |  |
|  |  | አይተላለፍም |  | 5 |
| 4 | መቸሊተላለፍ ይችላል | በርግዝና ወቅት | 1 |  |
|  |  | በወሊድ ወቅት | 2 |  |
|  |  | ጡት በማጥባጥ ወቅት | 3 |  |
|  |  | ሊላ |  |  |
|  |  |  |  |  |
| 5 | ከእናት ወደ ልጅ እንዳይተላለፍ መከላከል ይቻላል | አወ | 1 |  |
|  |  | ኤቻልም | 2 | 7 |
| 6 | ከእናት ወደ ልጅ እንዳይተላለፍ እንዲት መከላከል ይቻላል | ጸረ ኢች አይቪ | 1 |  |
|  |  | ክትባት | 2 |  |
|  |  | የባህል መዳኒት | 3 |  |
|  |  | ሊላ (ይጠቀስ ) |  |  |

**ክፍል ሶስት: ከእናት ወደ ልጅ እንዳይተላለፍ መከላከል እና እርግዝና**

| 1 | ኢች አይቪ ተመርምረሽ ታውቂያለሽ | አወ | 1 |  |
| --- | --- | --- | --- | --- |
|  |  | አላውቅም | 2 | 3 |
| 2 | ካልተመረመርሽ ለምን | ምረመራ መኖሩን አለማወቅ | 1 |  |
|  |  | ምርመራ ጣቢያ አለመኖር | 2 |  |
|  |  | ተጋላጸጭነት ስለለኝ | 3 |  |
|  |  | መመርመሩ ጥቅም ስለሊለው | 4 |  |
|  |  | ባለቢቲ ስለማይፈቅድልኝ | 5 |  |
|  |  | ምርመራውን ሚስጥረናነቱን ስለምጠራጠር | 6 |  |
| 3 | ለኢች አይቪ ተጋላጭነት አለኝ ብለሽታስቢያለሽ | አወ | 1 |  |
|  |  | አላስብም | 2 |  |
| 4 | ኢች አይቪ ውጢትሽ ኢች አይቪ መኖሩን ቢያሳይ ባለቢትሽ ወይም ቢተሰቦችሽ ምን ሊሉ ይችላሉ | ውጢቱን አይቀበሉትም | 1 |  |
|  |  | ከቢት ያሶጡኛል | 2 |  |
|  |  | ይደበደቡኛል | 3 |  |
|  |  | ይንከባከቡኛል | 4 |  |
